# Behavioral and social drivers of rotavirus vaccine uptake in a rural ethnic minority population in Vietnam: A cross-sectional study

**DOI:** 10.1016/j.pmedr.2025.103353

**Published:** 2025-12-18

**Authors:** Hung Huu Nguyen, Duc Tu Bui Ngo, Anh Ngoc Thi Nguyen, An Thu Thi Nguyen, Phuong Thu Thi Pham, Nguyen Lam Vuong

**Affiliations:** a Faculty of Public Health, University of Medicine and Pharmacy at Ho Chi Minh City, 217 Hong Bang, Cho Lon Ward, Ho Chi Minh City, Viet Nam; b School of Medicine, University of Medicine and Pharmacy at Ho Chi Minh City, 217 Hong Bang, Cho Lon Ward, Ho Chi Minh City, Viet Nam; c Oxford University Clinical Research Unit, 764 Vo Van Kiet, Cho Quan Ward, Ho Chi Minh City, Viet Nam

**Keywords:** Rotavirus vaccine, Vaccine uptake, Ethnic minority, Behavioral and social drivers

## Abstract

**Objective:**

This study aimed to assess rotavirus vaccine (RVV) intent/uptake among rural ethnic minority Vietnamese populations and identify the key drivers influencing vaccination decisions using the World Health Organization's Behavioral and Social Drivers of vaccination framework.

**Methods:**

We conducted a community-based cross-sectional study in a rural region of Vietnam in 2025. The study surveyed 384 mothers of children under five using a structured, interviewer-administered questionnaire. The primary outcome was RVV uptake/intent. Associated factors were explored using Poisson regressions, guided by a directed acyclic graph.

**Results:**

Only 9.4 % of children had received at least one dose of RVV, with a total of 16.9 % of mothers reporting either vaccine uptake or intent to vaccinate. Knowledge about rotavirus and vaccination was limited (median score of 3 out of a possible 7), while attitudes toward vaccination were favourable (median score of 12 out of 16). Higher knowledge and positive attitudes, social support from family, peers, community leaders, and healthcare workers were positively associated factors. Conversely, structural barriers such as geographic inaccessibility, and lack of communication were negative associated factors.

**Conclusions:**

RVV uptake among ethnic minority populations remains low. To close this gap, public health strategies must integrate culturally tailored communication, community-level engagement, and improved service accessibility.

## Highlights

• A total of 16.9% of mothers reported either vaccine uptake or intent to vaccinate.
• Knowledge about rotavirus and vaccination was generally low among mothers.
• Positive attitudes and social support promoted vaccine uptake or intent.
• Distance and poor infrastructure were key barriers to vaccination.
• Culturally tailored communication and access improvements are essential.

## 1 Introduction

Rotavirus infection is the leading global cause of acute gastroenteritis and severe diarrheal disease in children under five years old (GBD 2021 Diarrhoeal Diseases Collaborators, 2025; World Health Organization, 2025a). It accounts for about 15 % of all diarrheal deaths worldwide in 2021, and is responsible for an estimated 120,000 deaths and 10.8 million disability-adjusted life years (DALYs) from diarrhea annually (GBD 2021 Diarrhoeal Diseases Collaborators, 2025). Over 80 % of these deaths occur in sub-Saharan Africa, Southeast Asia, and South Asia (Tate et al., 2016), highlighting rotavirus-related gastroenteritis (RVGE) as a major public health issue, particularly in rural areas with poor sanitation and limited water access.

Immunization is considered one of the most effective strategies for controlling rotavirus disease (World Health Organization, 2021). Countries that have integrated the rotavirus vaccine (RVV) into their national immunization programs (NIPs) have seen a 36 % drop in RVGE-associated mortality among children under five (Burnett et al., 2020). Currently, four oral, live, attenuated RVVs are prequalified by the World Health Organization (WHO) (World Health Organization, 2021; Tahrat et al., 2025). In 2009, the WHO first recommended that all countries adopt RVVs into their NIPs, prioritizing regions like South and Southeast Asia and sub-Saharan Africa due to high RVGE fatality rates (World Health Organization, 2021). However, global RVV coverage remains suboptimal (Tahrat et al., 2025).

In Vietnam, the NIP provides free vaccinations to all children under five, independent of national health insurance coverage. The NIP includes ten vaccine-preventable diseases: hepatitis B, tuberculosis, diphtheria, pertussis, polio, measles, *Haemophilus influenzae* type B, Japanese encephalitis B, rubella, and tetanus. RVV was available on a self-pay basis until 2022, when the Ministry of Health planned to include it in the NIP. However, the nationwide rollout was delayed and ultimately halted due to vaccine supply chain disruptions caused by the COVID-19 pandemic. This delay, with the implementation still in an experimental phase as of 2024, has led to a number of preventable outbreaks, especially in vulnerable communities. For example, Ninh Thuan province, where ethnic minorities make up 23 % of the population, saw a 72.9 % increase in gastroenteritis cases, with 1224 new cases reported in the first nine months of 2023 (Ninh Thuan Statistical Office, 2023).

In resource-limited settings like Vietnam, there is a significant lack of data on RVV coverage since its planned introduction, particularly among ethnic minority populations. Assessing vaccine uptake is a crucial public health function. In 2022, the WHO introduced the Behavioral and Social Drivers (BeSD) framework to articulate the factors influencing vaccine uptake (World Health Organization, 2022). The framework groups determinants into four key domains: (1) thinking and feeling about vaccines; (2) social processes that drive or inhibit vaccination; (3) motivation to seek vaccination; and (4) practical issues involved in seeking and receiving vaccination. This study aims to gather data on RVV intent/uptake and to identify the primary drivers within a rural ethnic minority Vietnamese population. We selected Ninh Hai District, a coastal region of Ninh Thuan Province, as the study site because it includes diverse ethnic minority populations that are broadly representative of the province and is an area with a high risk of RVGE. This information will be vital for public health authorities and pediatricians, helping them to develop a more effective and impactful vaccination strategy to improve RVV uptake.

## 2 Methods

### 2.1 Study design and population

This was a community-based cross-sectional study conducted from February to April 2025 in Ninh Hai District, Ninh Thuan province, Vietnam. Ninh Hai District has a substantial ethnic minority population (approximately 20 %) and faces barriers such as limited access to healthcare and a high burden of diarrheal diseases. More than 30 ethnic minority groups reside within the district and the wider province, with the Cham and Raglai communities being the most prominent. The Raglai population, in particular, is among the poorest ethnic groups in Vietnam (Kozel, 2014). According to the 2019 census, the district has approximately 93,000 residents across nine commune-level subdivisions, with the local economy largely dependent on salt production and aquaculture.

The study protocol was approved by the Ethics Committee of the University of Medicine and Pharmacy at Ho Chi Minh City. All participants provided written informed consent prior to enrollment.

The target population for this study was mothers or primary female caregivers from ethnic minority households with at least one child under five years old. The required sample size was determined to estimate a single proportion with a 95 % confidence level and a 5 % margin of error. Based on a presumed RVV coverage prevalence of 35 % among children in the region, the calculated sample size was 366. Accounting for a 5 % non-response rate, the final target sample size was set at 384 participants.

A two-stage sampling procedure was used to recruit participants. First, two rural communes (Xuan Hai and Vinh Hai) were selected from the district's nine communes using a lottery method. In the second stage, participants were recruited from each of the two selected communes using convenience sampling through door-to-door visits until the required sample size of 192 participants per commune was reached.

Eligible participants were mothers or female caregivers aged 18 years or older, residing in one of the selected communes, and having at least one child under five years old. For households with multiple children meeting the criteria, the youngest child was selected for the study.

A total of 384 mothers with children under five years old were included in the study. The median maternal age was 31 years, and the median age of the children was 3.2 years. More than half of the mothers (51.5 %) reported a lack of fluency in Vietnamese, and educational attainment was low, with 51.3 % having a primary school education or below. The majority of households were classified as low-income (78.9 %), and most mothers (59.4 %) rarely or never used health insurance (Table 1).

**Table 1:** Sociodemographic characteristics of mothers from ethnic minority households in Ninh Hai District, Ninh Thuan Province, Vietnam, February to April 2025 (*N* = 384).

	Median (IQR) or n (%)
Age of mother (years)	31 (28, 36)
Age of child (years)	3.2 (1.8, 4.0)
Ethnicity	
Raglai	192 (50.0)
Cham	192 (50.0)
Religion	
None	192 (50.0)
Bani	168 (43.8)
Islam	24 (6.2)
Vietnamese proficiency	
Not fluent	198 (51.5)
Fluent	186 (48.5)
Education level	
Primary school diploma or lower	197 (51.3)
Secondary school diploma	50 (13.0)
High school diploma	90 (23.5)
Academic degree or higher	47 (12.2)
Household size	
1 to 3	120 (31.3)
4 or 5	204 (53.1)
More than 5	60 (15.6)
Household economic status	
Low	303 (78.9)
Middle	75 (19.5)
High	6 (1.6)
Health insurance usage	
Never/rarely	228 (59.4)
Regularly	156 (40.6)

IQR, interquartile range (25th; 75th percentiles).

### 2.2 Measures

Data from mothers or caregivers were collected using a structured, interviewer-administered questionnaire, developed based on the WHO BeSD framework (**Appendix 1**). The questionnaire, designed to be completed in about 15 min, was prepared in Vietnamese and included five sections: (1) sociodemographic characteristics; (2) thinking and feeling domain; (3) social processes domain; (4) practical issues domain; and (5) motivational and vaccine uptake domain. An English version of the questionnaire is available in the supplementary materials (**Appendix 2**).

Data collectors and supervisors underwent a two-day training session on data collection and sampling techniques. The questionnaire's acceptability and logical flow were checked during a pre-testing phase with 15 volunteers from the same ethnic minority groups in Ninh Hai District. These responses were not included in the main analysis. To ensure accuracy, child vaccination data was primarily collected from the child's immunization card. If a card was unavailable, data was gathered through a direct interview with the parent or caregiver. Field investigators conducted daily checks for completeness and quality to maintain data integrity.

RVV was administered orally, with at least four weeks between doses. The recommended schedule for full vaccination was a two-dose series given at 6 and 14 weeks of age. The main outcome of interest was the motivational aspect of RVV uptake or intent to vaccinate among parents and caregivers. This was a binary variable (yes/no) defined as “yes” if the child had received at least one dose of RVV or if the parent/caregiver expressed an intention to have their child vaccinated. The intent to vaccinate was assessed with the question, “If you could satisfy all necessary conditions, would you rather your child be vaccinated against rotavirus?”

Independent variables included sociodemographic characteristics and factors grouped into three domains based on the WHO BeSD framework: “thinking and feeling”, “social processes”, and “practical issues”.

The “thinking and feeling” domain was measured to evaluate rotavirus-specific perception and confidence in RVV. The rotavirus-specific perception (i.e., knowledge) was assessed through four items related to maternal knowledge of RVV utility (maximum 1 point), RVGE prevention (maximum 4 points), complications (maximum 1 point), and transmission (maximum 1 point). A total knowledge score, ranging from 0 to 7, was calculated, with a higher score indicating a better understanding of rotavirus and RVV. The confidence (i.e., attitude) was measured on a 4-point Likert scale, evaluating agreement with statements on the commonness of rotavirus, necessity of RVV, need for vaccination information, and general need for vaccination. The total attitude score ranged from 4 to 16, with a higher score representing a more positive attitude toward rotavirus vaccination.

The “social processes” domain was assessed using six variables related to social experiences, including religious leader norms, community leader norms, peer norms, family norms, mother's autonomy and healthcare workers recommendation regarding RVV.

The “practical issues” domain was evaluated by assessing six barriers to RVV uptake among ethnic minority groups, including knowledge of vaccination sites, service quality, service dissatisfaction details, financial barrier, road condition, and distance to the nearest health service.

### 2.3 Statistical analysis

Descriptive statistics were used to summarize the data, reporting continuous variables as median with interquartile range (IQR) and categorical variables as frequency and percentage. Factors associated with the main outcome of RVV uptake or intent were first explored using univariable Poisson regression models with robust standard errors. Results from these models were reported as prevalence ratios (PRs) with 95 % confidence intervals (CIs).

**Fig. 1:**
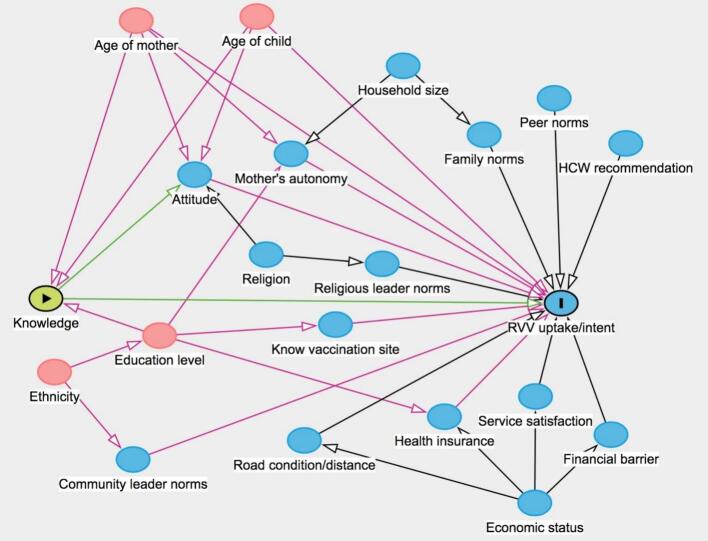
Assumptions about the causal relationships between factors and rotavirus vaccine uptake or intent among mothers from ethnic minority households in Ninh Hai District, Ninh Thuan Province, Vietnam, February to April 2025. The graph was developed using https://www.dagitty.net/. In the directed acyclic graph, the green node (Knowledge) represents the main exposure of interest, and the blue node with an ‘I' indicates the outcome of interest (RVV uptake/intent). Pink nodes represent ancestors of both the exposure and the outcome (i.e., confounding factors), while blue nodes represent ancestors of the outcome only. Green arrows indicate the causal path from exposure to outcome; pink arrows represent biasing paths; and black arrows indicate paths that are neither causal nor biasing. RVV, rotavirus vaccine. (For interpretation of the references to colour in this figure legend, the reader is referred to the web version of this article.)

We developed a directed acyclic graph (DAG) to visually display assumptions about the causal relationships between the sociodemographic characteristics, the factors from the BeSD framework, and the outcome of interest (Fig. 1). Our analysis specifically focused on the causal pathway from participants' knowledge and attitude to RVV uptake/intent. Potential confounders that were identified and controlled for included age of mother, age of child, household size, health insurance usage, community leader norms, mother's autonomy, knowledge of vaccination sites, service satisfaction, financial barrier, and road condition and distance. Finally, a multivariable Poisson regression model with robust standard errors was employed to estimate the adjusted effects, including knowledge and all identified confounders. All statistical analyses were performed using Stata version 17 (College Station, TX, USA).

## 3 Results

### 3.1 BeSD framework indicators

In the “thinking and feeling” domain, knowledge about rotavirus and vaccination was limited, with a median score of 3 (IQR: 1, 4) out of a possible 7. In contrast, attitudes toward vaccination were generally favourable, with a median score of 12 (IQR: 10, 14) out of 16 (Table 2).

**Table 2:** Factors based on the Behavioral and Social Drivers framework among mothers from ethnic minority households in Ninh Hai District, Ninh Thuan Province, Vietnam, February to April 2025 (N = 384).

	Median (IQR) or n (%)
***Thinking and feeling domain***	
Knowledge of rotavirus and RV vaccination	3 (1, 4)
Attitude of rotavirus vaccination	12 (10, 14)
***Social processes domain***	
Religious leader norms	308 (80.2)
Community leader norms	244 (63.5)
Peer norms	285 (74.2)
Family norms	313 (81.5)
Mother's autonomy	242 (63.0)
Health workers recommendation	121 (31.5)
***Practical issues domain***	
Know vaccination site	372 (96.9)
Overall service satisfaction	379 (99.0)
Details of service dissatisfaction	
Long waiting time	157 (41.0)
Vaccine not always available	111 (29.0)
Staff do not speak ethnic language	96 (25.0)
Not enough time with clients	16 (4.2)
Clinic does not open on time	1 (0.3)
Biggest financial barrier for vaccination (*N* = 383)⁎	
Service expense	104 (27.2)
Travel cost	82 (21.4)
Time cost for travel	74 (19.3)
Adverse event cost	74 (19.3)
Time cost post-vaccination	49 (12.8)
Inconvenient road infrastructure	185 (48.2)
Distance to the nearest health service (km)	4.0 (3.5, 6.0)
***Motivational domain and RVV uptake***	
Received at least one dose of RVV	36 (9.4)
RVV uptake or intent of RVV uptake	65 (16.9)

IQR, interquartile range (25th; 75th percentiles); RVV, rotavirus vaccine.

⁎ One participant had never used vaccination service.

Within the “social processes” domain, the majority of participants reported normative support for vaccination from their family (81.5 %), religious leaders (80.2 %), and peers (74.2 %). Additionally, 63 % of mothers reported a high degree of autonomy in their vaccination decisions. However, only 31.5 % reported receiving a vaccination recommendation from healthcare workers.

In the “practical issues” domain, nearly all mothers knew where to access vaccination services (96.9 %), and almost all expressed overall satisfaction with the service (99 %). Despite this, participants reported several barriers, including long waiting times (41 %), unavailability of vaccines (29 %), and a lack of communication in their ethnic language (25 %). The biggest financial barriers were service costs (27.2 %) and travel expenses (21.4 %). Nearly half of the participants reported poor road conditions to the vaccination center. The median distance to the nearest health facility was 4 km (IQR: 3.5, 6).

Overall, only 9.4 % of children in the study had received at least one dose of RVV, while 16.9 % of mothers expressed either vaccine uptake or the intent to vaccinate.

### 3.2 Associations with RVV uptake/intent

Younger child age was significantly associated with a higher likelihood of RVV uptake/intent (PR [95 % CI]: 0.54 [0.44, 0.66] per 1-year increase). Regarding ethnicity, being Cham was strongly associated with a higher likelihood of uptake/intent compared to being Raglai (PR: 15.25 [5.65, 41.16]). Other positively associated factors included fluency in Vietnamese (PR: 10.47 [4.63, 23.69]), higher maternal education (PR: 7.32 [3.08, 17.40], 7.50 [3.35, 16.79], and 12.57 [5.68, 27.83] for secondary school, high school, and academic degree compared with primary school or lower, respectively), larger household size (PR: 4.59 [1.86, 11.33] and 8.4 [3.33, 21.19] for 4–5 and > 5 members vs. 1–3), higher income (PR: 6.24 [2.62, 14.84] for high vs. low), and regular use of health insurance (PR: 7.17 [3.87, 13.29]) (Table 3).

**Table 3:** Association between sociodemographic characteristics and rotavirus vaccine uptake or intent among mothers from ethnic minority households in Ninh Hai District, Ninh Thuan Province, Vietnam, February to April 2025.

Factor	PR	95 % CI
Age of mother (years)	1.03	1.00, 1.07
Age of child (years)	0.54	0.44, 0.66
Ethnicity		
Raglai	1	Ref
Cham	15.25	5.65, 41.16
Religion		
None	1	Ref
Bani	16.00	5.92, 43.25
Islam	10.00	2.88, 34.77
Vietnamese proficiency		
Not fluent	1	Ref
Fluent	10.47	4.63, 23.69
Education level		
Primary school diploma or lower	1	Ref
Secondary school diploma	7.32	3.08, 17.40
High school diploma	7.50	3.35, 16.79
Academic degree or higher	12.57	5.68, 27.83
Household size		
1 to 3	1	Ref
4 or 5	4.59	1.86, 11.33
More than 5	8.40	3.33, 21.19
Household economic status		
Low	1	Ref
Middle	1.64	0.81, 3.31
High	6.24	2.62, 14.84
Health insurance usage		
Never/rarely	1	Ref
Regularly	7.17	3.87, 13.29

CI, confidence interval; PR, prevalence ratio; Ref, reference.

Among the BeSD indicators, both higher knowledge scores (PR: 1.83 [1.57, 2.14] per 1-point increase) and more favourable attitudes (PR: 1.44 [1.30, 1.61] per 1-point increase) were significantly associated with RVV uptake/intent. All “social processes” factors showed a positive association: religious leader norms (PR: 5.1 [1.64, 15.84]), community leader norms (PR: 11.86 [3.79, 37.12]), peer norms (PR: 10.94 [2.72, 43.98]), family norms (PR: 4.69 [1.51, 14.55]), mother's autonomy (PR: 3.65 [1.86, 7.16]), and healthcare workers recommendation (PR: 15.5 [7.62, 31.46]) (Table 4).

**Table 4:** Association between factors based on the Behavioral and Social Drivers framework and rotavirus vaccine uptake or intent among mothers from ethnic minority households in Ninh Hai District, Ninh Thuan Province, Vietnam, February to April 2025.

Factor	PR	95 % CI
***Thinking and feeling domain***		
Knowledge of rotavirus and RVV score	1.83	1.57, 2.14
Attitude of rotavirus vaccination score	1.44	1.30, 1.61
***Social processes domain***		
Religious leader norms	5.10	1.64, 15.84
Community leader norms	11.86	3.79, 37.12
Peer norms	10.94	2.72, 43.98
Family norms	4.69	1.51, 14.55
Mother's autonomy	3.65	1.86, 7.16
Health workers recommendation	15.50	7.62, 31.46
***Practical issues domain***		
Know vaccination site	2.06	0.31, 13.69
Overall service satisfaction	0.22	0.12, 0.40
Details of service dissatisfaction		
Long waiting time	1.09	0.70, 1.70
Vaccine not always available	1.74	1.12, 2.71
Staff do not speak ethnic language	0.25	0.10, 0.61
Not enough time with clients	1.11	0.39, 3.16
Biggest financial barrier for vaccination		
Service expense	1	Ref
Travel cost	0.54	0.26, 1.12
Time cost for travel	0.67	0.33, 1.34
Adverse event cost	1.07	0.60, 1.91
Time cost post-vaccination	0.91	0.45, 1.84
Inconvenient road infrastructure	1.11	0.71, 1.73
Distance to the nearest health service (km)	0.88	0.81, 0.96

CI, confidence interval; PR, prevalence ratio; Ref, reference; RVV, rotavirus vaccine.

Within the “practical issues” domain, the data revealed some notable associations. Surprisingly, overall service satisfaction showed a negative association with RVV uptake/intent (PR: 0.22 [0.12, 0.40]). In contrast, mothers reporting vaccine unavailability were more likely to express uptake /intent (PR: 1.74 [1.12, 2.71]), while a lack of ethnic language communication reduced this likelihood (PR: 0.25 [0.10, 0.61]). Greater distance to the nearest health facility was also negatively associated with uptake/intent (PR: 0.88 [0.81, 0.96] per km) (Table 4).

### 3.3 Multivariable analysis

In the adjusted model, knowledge score (PR [95 % CI]: 1.55 [1.23, 1.96] per 1-score increase), younger child age (PR: 0.58 [0.48, 0.69] per 1-year increase), and regular health insurance use (PR: 2.02 [1.08, 3.79]) remained significantly positively associated factors with RVV uptake/intent. Service satisfaction was also positively associated (PR: 3.73 [1.14, 12.23]). Conversely, elevated opportunity costs, including time and travel (PR: 0.40 [0.22, 0.74]) and greater distance to the nearest facility (PR: 0.69 [0.55, 0.87] per km) continued to negatively affect uptake/intent (Table 5).

**Table 5:** Multivariable analysis of factors associated with rotavirus vaccine uptake or intent among mothers from ethnic minority households in Ninh Hai District, Ninh Thuan Province, Vietnam, February to April 2025.

Factor	PR	95 % CI
Knowledge of rotavirus and RVV score	1.55	1.23, 1.96
Age of mother (years)	0.99	0.96, 1.03
Age of child (years)	0.58	0.48, 0.69
Household size		
1 to 3	1	Ref
4 or 5	1.87	0.81, 4.36
More than 5	2.01	0.75, 5.37
Health insurance usage (regularly)	2.02	1.08, 3.79
Community leader norms	1.07	0.81, 1.42
Mother's autonomy	1.52	0.87, 2.63
Know vaccination site	0.51	0.08, 3.40
Overall service satisfaction	3.73	1.14, 12.23
Biggest financial barrier for vaccination		
Service expense	1	Ref
Travel cost	0.56	0.31, 1.03
Time cost for travel	0.40	0.22, 0.74
Adverse event cost	0.72	0.42, 1.22
Time cost post-vaccination	0.54	0.30, 0.97
Inconvenient road infrastructure	0.78	0.53, 1.14
Distance to the nearest health service (km)	0.69	0.55, 0.87

CI, confidence interval; PR, prevalence ratio; Ref, reference; RVV, rotavirus vaccine.

## 4 Discussion

Our study identified key behavioral and social drivers of RVV uptake and intent among ethnic minority mothers in Vietnam. Only 9.4 % of children had received at least one RVV dose, and just 16.9 % of mothers reported vaccinating or intending to vaccinate their child.

By the end of 2024, RVVs had been integrated into the national immunization programs of 131 countries, with global coverage around 59 % (World Health Organization, 2025b). In Vietnam, the National Expanded Program on Immunization reported over 297,000 free doses administered by May 2025 across 41 provinces, where more than 90 % of eligible children were vaccinated on time (UNICEF, 2025). Compared with these figures, uptake in our study area remains considerably lower, revealing persistent disparities in access and acceptance among ethnic minority populations. These inequities likely reflect linguistic, cultural, and geographic barriers that limit healthcare access, not only for RVV but also for other vaccines. Early RVV adoption in countries such as the United States, India, Brazil, and across Europe led to much higher coverage (World Health Organization, 2025c; Ghaswalla et al., 2021; Raju et al., 2019; de Oliveira Roque et al., 2023), underscoring the importance of national commitment and equitable implementation. The gap observed in our study highlights the urgent need to address both systemic and behavioral barriers to improve RVV uptake in minority communities.

Several important determinants of vaccine uptake were identified. Younger child age was positively associated with RVV uptake, a finding consistent with previous studies indicating that parental willingness to vaccinate decreases as children grow older (Rees et al., 2022). Ethnicity, language proficiency, and educational attainment also emerged as strong predictors, underscoring the role of cultural and communication barriers in vaccination decisions (La Fauci et al., 2024; Tu et al., 2023). Different ethnic groups were associated with varying likelihoods of vaccination due to differences in economic status and living conditions; those with higher income and better living conditions tend to have a higher proportion of RVV intent/uptake. These findings suggest that interventions must go beyond individual-level education and address broader socio-cultural determinants of health. Practical barriers were also prominent. Distance to healthcare facilities and poor road infrastructure reduced the likelihood of uptake, as reported in other low-to-middle-income countries (LMICs) (de Oliveira Roque et al., 2023; Osborne et al., 2025). Interestingly, mothers who reported vaccine unavailability were more likely to express vaccination intent, possibly reflecting heightened demand or frustration with service delivery. Conversely, lack of communication in ethnic minority languages significantly hindered uptake, underscoring the need for culturally and linguistically tailored health services.

The BeSD framework provided useful insights into cognitive, social, and structural influences on vaccine behavior. Other studies also found that low uptake of RVVs was linked to low perceived preventive value, concerns about side effects, limited literacy, and systemic healthcare limitations – barriers aligning closely with BeSD domains of knowledge, attitudes, trust, and access (Casigliani et al., 2022; Biset et al., 2021; Kadambari and Vanderslott, 2021; Lorini et al., 2018; Zhang et al., 2023). Social norms played a particularly influential role, with support from family, peers, and community leaders strongly associated with vaccination intent, similar to other studies (La Fauci et al., 2024; McDermid et al., 2024). Consistent with global evidence, healthcare worker recommendations proved to be the most powerful driver (Karras et al., 2024; Birkhäuer et al., 2017), highlighting the importance of trusted voices in promoting vaccine acceptance. These parallels reinforce that despite contextual differences, BeSD framework domains consistently shape vaccination behavior–affirming the relevance and generalizability of our findings. It underscores the pressing need for interventions that simultaneously address cognitive (knowledge and attitudes), social (trusted communication and norms), and structural (access, logistics, equity) determinants to successfully increase vaccine uptake among marginalized communities.

The findings of this study underscore the urgent need for equity-oriented strategies to improve RVV uptake among ethnic minorities in Vietnam. First, health communication must be culturally and linguistically tailored, ensuring that caregivers receive information in their native languages and in ways that respect community traditions and beliefs. Second, strengthening the role of healthcare workers and community leaders as trusted messengers is essential. Third, equally important is addressing structural barriers by ensuring reliable vaccine supply, reducing geographic and financial obstacles, and improving transportation infrastructure to facilitate access to immunization services. Finally, integrating RVVs more fully into the NIP with a specific focus on disadvantaged populations will be critical for closing existing coverage gaps. Together, these multi-level interventions—encompassing social, behavioral, and structural approaches—can substantially enhance vaccine coverage and reduce health inequities in these communities.

We acknowledge several limitations of our study. Firstly, the cross-sectional design limits causal inference and prevents investigation of the relationship between motivation (intent to vaccinate) and practice (RVV uptake). Secondly, reliance on self-reported vaccination history may introduce recall bias. Finally, the study was conducted in a single province and may not fully represent other ethnic minority populations in Vietnam or in other LMICs.

In conclusion, RVV uptake among ethnic minority groups in Vietnam remains low, shaped by socio-demographic, behavioral, and structural factors. Limited knowledge, language barriers, and poor access impede uptake, while social support and healthcare worker recommendations encourage it. Culturally tailored communication, community engagement, and better service access—along with equitable vaccine supply and national program integration—are essential to improve coverage and reduce preventable diarrheal disease.

## CRediT authorship contribution statement

**Hung Huu Nguyen:** Writing – review & editing, Writing – original draft, Methodology, Investigation, Formal analysis, Conceptualization. **Duc Tu Bui Ngo:** Writing – review & editing, Writing – original draft, Conceptualization. **Anh Ngoc Thi Nguyen:** Writing – review & editing, Writing – original draft, Investigation. **An Thu Thi Nguyen:** Writing – review & editing, Writing – original draft, Investigation. **Phuong Thu Thi Pham:** Writing – review & editing, Writing – original draft, Investigation. **Nguyen Lam Vuong:** Writing – review & editing, Writing – original draft, Methodology, Formal analysis, Conceptualization.

## Ethics approval and consent to participate

The study protocol was approved by the Ethics Committee of the University of Medicine and Pharmacy at Ho Chi Minh City (No. 197/ĐHYD-HĐĐĐ, dated January 9, 2025) and was conducted in accordance with the principles of the Declaration of Helsinki. All participants provided written informed consent prior to enrollment. To guarantee data confidentiality, all information was secured using a coding system and stored in a locked cabinet.

## Funding

This research was funded by the University of Medicine and Pharmacy at Ho Chi Minh City under contract number 177/2025/HĐ-ĐHYD, dated April 17, 2025.

## Declaration of competing interest

All authors have no competing interests to declare.

## Data Availability

The datasets used and/or analyzed during the current study are available from the corresponding author on reasonable request.

## Appendix A Supplementary data

Supplementary material Image 1
