# Supplementary material for: Behavioral and social drivers of rotavirus vaccine uptake in a rural ethnic minority population in Vietnam: A cross-sectional study
Source: Prev Med Rep. 2025 Dec 18;61:103353. doi: 10.1016/j.pmedr.2025.103353 (PMC12808582; doi:10.1016/j.pmedr.2025.103353)
Supplement: Supplementary file 1 — Supplementary material [file mmc1.docx]

**Appendix 1. Behavioural and Social Drivers framework of rotavirus vaccine uptake among mothers from ethnic minority households in Ninh Hai District, Ninh Thuan Province, Vietnam, February to April 2025**

**
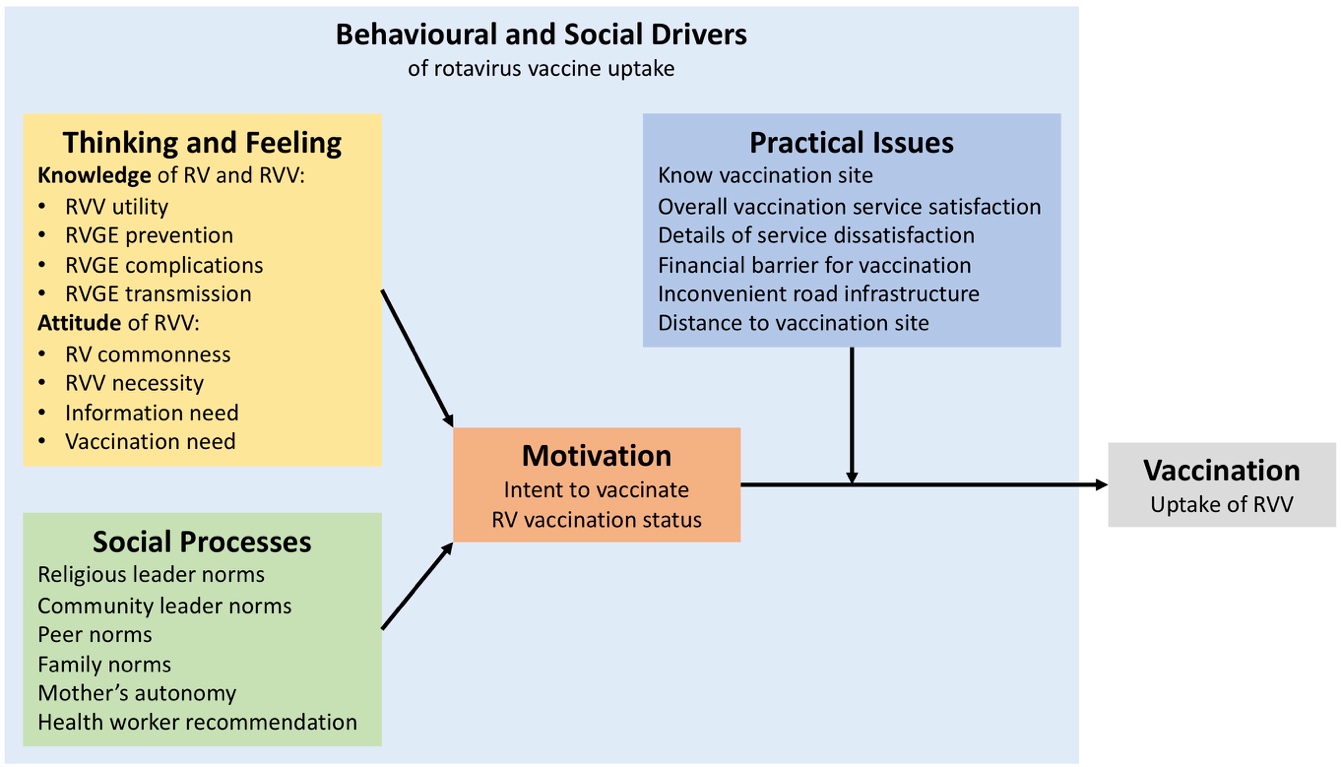
**

RV, rotavirus; RVV, rotavirus vaccines; RVGE, rotavirus gastroenteritis

**Appendix 2. Questionnaire for interviewing mothers from ethnic minority households in Ninh Hai District, Ninh Thuan Province, Vietnam, February to April 2025**

**General information**

- ID of questionnaire:
- Name of interviewer:
- Date of interview:

**Section 1: Sociodemographic characteristics**

| **Variable** | **Question** | **Coding** | **Response** |
| --- | --- | --- | --- |
| Age of mother | How old are you? |  | …………………………… years |
| Age of child | What is your youngest child’s date of birth? |  | …………../………../………………..  (DD/MM/YYYY) |
| Ethnicity | What ethnic group do you belong to? | 0  1  2 | Cham  Raglai  Other (specify): ……………………. |
| Religion | What religious group do you belong to? | 0  1  2  3  4  5 | Catholicism  Protestantism  Buddhism  Bani  Islam  Other (specify): ……………………... |
| Vietnamese proficiency | How fluent are you in Vietnamese? | 0  1  2 | Do not understand  Partially understand  Completely understand |
| Education level | What is the highest education level you completed? | 1  2  3  4  5 | Lower than primary school  Primary  Secondary  High school  Academic degree or higher |
| Household size | How many people usually live in your household? |  | ……………………………………people |
| Household economic status | How would you evaluate your household’s economic status? | 0  1  2 | Low  Middle  High |
| Health insurance usage | How often do you use your health insurance? | 0  1  2 | Never/Rarely  Sometimes  Regularly |

**Section 2: Knowledge and attitudes toward immunization (thinking and feeling domain)**

| **Variable** | **Question** | **Coding** | **Response** |
| --- | --- | --- | --- |
| ***Rotavirus-specific perceptions*** | | | |
| RVV utility | What disease does the Rotavirus vaccine prevent? | 0  1  2  3  4 | Rotavirus gastroenteritis  Hepatitis  Pneumonia  Influenza  Unknown |
| RVGE prevention | How can you prevent your child from gastroenteritis?  (*Multiple choices*) | 0  1  2  3  4 | Breastfeeding  Vaccination  Boiled water  Handwashing  Unknown |
| RVGE complications | Can rotavirus gastroenteritis be fatal to your child? | 1  0 | Yes  No |
| RVGE transmission | Can rotavirus gastroenteritis be transmitted person-to-person? | 1  0 | Yes  No |
| ***Rotavirus vaccine confidence*** | | | |
| Rotavirus commonness | “Rotavirus gastroenteritis is common in children and non-fatal.” | 4  3  2  1 | Strongly disagree  Disagree  Agree  Strongly agree |
| RVV necessity | “Preventing rotavirus gastroenteritis is not necessary.” | 4  3  2  1 | Strongly disagree  Disagree  Agree  Strongly agree |
| Information need | I would like to receive more information about rotavirus vaccine? | 1  2  3  4 | Strongly disagree  Disagree  Agree  Strongly agree |
| Vaccination need | I want my child to be vaccinated against rotavirus despite possible risks. | 1  2  3  4 | Strongly disagree  Disagree  Agree  Strongly agree |

**Section 3: Social and cultural influences (social processes domain)**

| **Variable** | **Question** | **Coding** | **Response** |
| --- | --- | --- | --- |
| Religious leader norms | Do religious leaders support child vaccination? | 1  0 | Yes  No |
| Community leader norms | Do community leaders support child vaccination? | 1  0 | Yes  No |
| Peer norms | Do most parents you know vaccinate their children? | 1  0 | Yes  No |
| Family norms | Do most family members support child vaccination? | 1  0 | Yes  No |
| Mother’s autonomy | Does the mother need permission to take the child to clinic for vaccination? | 1  0 | Yes  No |
| Health worker recommendation | Has a health worker recommended rotavirus vaccine for your child? | 1  0 | Yes  No |

**Section 4: Practical barriers to vaccination (practical issues domain)**

| **Variable** | **Question** | **Coding** | **Response** |
| --- | --- | --- | --- |
| Know vaccination site | Do you know where to take your child for vaccination? | 1  0 | Yes  No |
| Overall service satisfaction | Overall, how satisfied are you with the vaccination experience? | 0  1 | Not satisfy  Partially/fully satisfied |
| Service dissatisfaction | What is not satisfactory about the services? (*Multiple choices*) | 1  2  3  4  5  6  7  8  0  9 | Vaccine not always available  Clinic does not open on time  Long waiting time  Center not clean  Staff not competent  Staff have poor attitude  Not enough time with clients  Staff do not speak ethnic language  Completely satisfied  Other (specify): ………………………….  ………………………….…………………………. |
| Biggest financial barrier | Which of the following is the biggest burden for your household?  (*Multiple choices*) | 0  1  2  3  4 | Service expense  Travel cost  Time cost for travel  Time cost post-vaccination  Adverse event cost |
| Road infrastructure | What is the road condition to the nearest clinic? | 0  1  2  3 | Impassable trail  Trail with vehicle access  Dirt road  Concrete/asphalt road |
| Distance | How far is the nearest health service? |  | ………………… km |

**Section 5: Vaccination status and intent (Motivational domain and RVV uptake)**

| **Variable** | **Question** | **Coding** | **Response** |
| --- | --- | --- | --- |
| Rotavirus vaccination status | Is your child vaccinated with rotavirus vaccine? (*Check card/record*) | 0  1 | Vaccinated  Not vaccinated |
| Intent to vaccinate | If all conditions are met, would you vaccinate your child against rotavirus? | 0  1 | Yes  No |
